# Advances toward precision therapeutics for developmental and epileptic encephalopathies

**DOI:** 10.3389/fnins.2023.1140679

**Published:** 2023-04-06

**Authors:** Ilaria Bertocchi, Marco Cambiaghi, Mazahir T. Hasan

**Affiliations:** ^1^Laboratory of Neuropsychopharmacology, Department of Neuroscience Rita Levi Montalcini, Institute of Neuroscience Cavalieri Ottolenghi (NICO), University of Turin, Torino, Italy; ^2^Department of Neuroscience Rita Levi Montalcini, Neuroscience Institute of Turin (NIT), Torino, Italy; ^3^Department Neuroscience, Biomedicine and Movement Sciences, University of Verona, Verona, Italy; ^4^Laboratory of Brain Circuits Therapeutics, Achucarro Basque Center for Neuroscience, Leioa, Spain; ^5^Ikerbasque – Basque Foundation for Science, Bilbao, Spain

**Keywords:** epilepsy, rodent models, therapeutics, adeno-associated virus, rare diseases

## Abstract

Developmental and epileptic encephalopathies are childhood syndromes of severe epilepsy associated with cognitive and behavioral disorders. Of note, epileptic seizures represent only a part, although substantial, of the clinical spectrum. Whether the epileptiform activity *per se* accounts for developmental and intellectual disabilities is still unclear. In a few cases, seizures can be alleviated by antiseizure medication (ASM). However, the major comorbid features associated remain unsolved, including psychiatric disorders such as autism-like and attention deficit hyperactivity disorder-like behavior. Not surprisingly, the number of genes known to be involved is continuously growing, and genetically engineered rodent models are valuable tools for investigating the impact of gene mutations on local and distributed brain circuits. Despite the inconsistencies and problems arising in the generation and validation of the different preclinical models, those are unique and precious tools to identify new molecular targets, and essential to provide prospects for effective therapeutics.

## 1. Introduction

**Figure 1:**
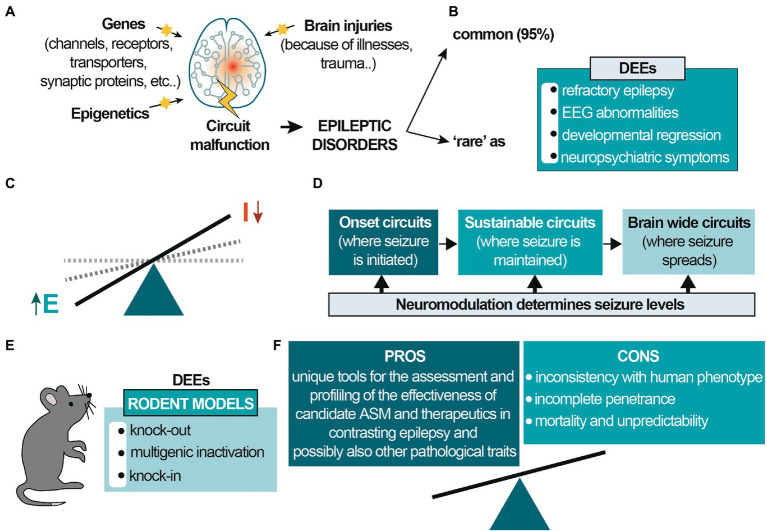
Etiology and circuit-based malfunctions in epilepsy. **(A)** Different genes, environmental/epigenetic factors and brain injuries may be the cause of circuit malfunctions that give rise to epilepsy, a network disease involving different interacting brain regions; **(B)** the prevalence of epileptic diseases is classified into common and rare epilepsies (~95 and ~5%, respectively). Developmental and epileptic encephalopathies (DEEs) are rare diseases mainly occurring in pediatric age. **(C)** In epileptic disorders the excitatory drive increases, generating hyperexcitability and E/I dysbalance; **(D)** epilepsy is initiated in different brain regions and activates synaptic brain circuits for seizure maintenance. It can further spread to other brain regions, generating comorbidities and increasing seizure severity. **(E)** Genetic rodent models for DEE mainly consist of knockout, multigenic deletions, and knock-in. These latter may be based on precise human point mutations, satisfying the criteria for ‘construct validity completely’; **(F)** Different pros and cons characterize these animal models. The most significant caveats concern the frequent inconsistencies with the human phenotype, particularly the possible absence of the epileptic phenotype and the incomplete penetrance of the mutation. The few models showing spontaneous epileptic seizures are often not viable or affected by a high mortality rate, and the seizures’ unpredictability makes them difficult to study. Despite this, genetic animal models are unique and valuable tools for assessing new candidate ASM and other therapeutic strategies and are necessary for subsequent clinical studies, thus benefits prevail.

A large battery of genes, epigenetic changes, and environmental factors such as pollutants, diet, or brain injuries are key factors that modify brain circuits to generate epileptic disorders that involve multiple interconnected brain regions for epilepsy onset and development (Figure 1A). Brain modifications may result in the alteration of the fine balance between excitation and inhibition (E/I balance; Figure 1C), and a seizure can arise in different brain areas and further spreads to other synaptically-connected brain regions, increasing its severity (Figure 1D).

Worldwide, the prevalence of epileptic diseases is classified into common and rare epilepsies, representing ~95 and ~5%, respectively. Developmental and epileptic encephalopathies (DEEs) are rare diseases (Figure 1B) and comprise a large, heterogeneous group of devastating epileptic disorders occurring in pediatric age. Those are mainly characterized by pharmaco-resistant polymorphous epilepsy, severe electroencephalogram (EEG) abnormalities, and developmental regression (Lin et al., 2022). Patients experience a broad spectrum of neuropsychiatric symptoms ranging from less to more severe, including neurological impairments, intellectual disability, sensory and communication deficits, and other significant psychiatric, motor, and behavioral alterations. DEEs are caused by various factors, among which 30–50% is genetic in origin, primarily due to sporadic *de novo* mutations affecting genes involved in the control of neuronal excitability, mainly genes encoding synaptic proteins and ion channels (Allen et al., 2016; von Deimling et al., 2017; Oyrer et al., 2018).

Recent technological advances, among which next-generation whole exome sequencing, have identified many variants. However, due to continuous new diagnoses and mapping of genetic profiles, the number of causative genes for DEEs is continuously growing (McTague et al., 2016; Bayat et al., 2021; Platzer et al., 2022). Despite these progresses, there are no effective therapies approved explicitly for DEEs. Treatment is only symptomatic, aimed at controlling epileptic seizures and restoring abnormal electrical brain activity to reduce the short and long-term sequelae of DEEs. Still, even with polytherapy, partial or complete recovery is rare.

The diagnosis, treatment, and search for a cure for the different DEEs are further complicated by several factors, considering that few cases are currently described and that the patients present variable clinical pictures with multiple comorbidities. Hence, defining an optimal therapy for the various forms of DEEs is still a huge challenge. At the same time, the burden for patients and families remains significant, considering the potential risk of premature mortality, including sudden death (Radaelli et al., 2018). Moreover, one age-related epilepsy syndrome may sometimes evolve into another over time, resulting in many patients with phenotypes not fitting into specific syndromes. Indeed, it is worth considering that mutations in individual genes may give rise to a broad range of phenotypes, and different genes may cause the same syndrome. Many factors contribute to phenotypic heterogeneity, including type and temporal expression of the mutation, mosaicism, convergence of mechanistic pathways, and genome regulators such as epigenetic factors and modifier genes, leading to difficulties in providing genotype/phenotype correlations (Pisani et al., 2018; Guerrini et al., 2023). The complexity of such a scenario makes it clear that a great deal of knowledge is still to be investigated.

Given the phenotypic pleiotropy of gene mutations and the individual heterogeneity of drug responses, a precision medicine targeting the underlying pathology can potentially treat the overall symptomatology and may be considered the most beneficial for DEE patients (Pisani et al., 2018; Raga et al., 2021). Nowadays, novel advanced precision medicine approaches, such as antisense oligonucleotides (ASOs) and other gene therapy strategies (viral vector systems and CRISPR/Cas9 technology), have raised excitement within the research and medical communities and patients toward effective treatments of rare genetic diseases (Tang et al., 2021). Identifying several causative genes for DEEs has undoubtedly been a tremendous advantage for generating specific genetic animal models. Developing and validating novel preclinical models is fundamental to better understand the general molecular mechanisms that can be common to the different syndromes. Furthermore, the results obtained by testing novel therapies on animal models may help to understand which symptoms’ domains are expected to be affected by the new treatment when translated into the clinic.

This minireview briefly focuses on the challenges and opportunities of preclinical genetic models for DEEs, considering a few of them as an example.

Moreover, we will mention some crucial targets for possible circuit-based innovative tools for therapy.

## 2. Tools and targets

### 2.1. Rodent models for DEEs: Merits and caveats

There are over a hundred validated DEE-associated genes, the more significant portion including genes that code ion channels or ionotropic receptor subunits (for an exhaustive review, also about animal models, see Guerrini et al., 2023). Despite this, the number is increasing, considering the syndromes’ complexity and problematic categorization. Genetically modified rodent models for DEEs consist of classic and conditional knockout, multigenic deletions, and knock-in rodents (Figure 1E). These latter can be based on precise human point mutations, reproducing faithfully the genetic cause responsible for human monogenic disorders. However, the phenotypic spectrum caused by variants in monogenic epilepsy genes is broad and of varying severity, and the mechanisms leading to pleiotropy is not well understood. On one side, the innovative CRISPR-Cas9 technology provides enormous possibilities to quickly generate murine models carrying “patient-specific” mutations for preclinical investigation (Tang et al., 2021). On the other, despite satisfying the criteria for “construct validity,” these models still present many caveats, mainly concerning “face validity” (Turner et al., 2021).

First, some of the genetic rodent models generated so far do not show epileptic activity, e.g., Cdkl5 gene knockout or mutated mice (Fallah and Eubanks, 2020) or most of the viable mouse models carrying mutation/deletion in one of the genes coding for the different NMDA receptor subunits: *Grin1*, *Grin2a*, *Grin2b*, or *Grin2d* (Oyrer et al., 2018). These last rodent models are generated to mimic *GRIN*-related disorders, also called “grinpathies” (Santos-Gómez et al., 2021). Compared to rodent models, the alterations of *GRIN* genes in humans have a much more important impact, considering that they are often dominant (XiangWei et al., 2018). Only recently, two mutant mouse models with gain-of-function variants on the GluN2A subunit, which is frequently associated with epilepsy in humans (XiangWei et al., 2018), showed an epileptic phenotype and other symptoms similar to the ones described in DEE patients carrying analogous mutations, becoming, therefore, valuable mouse models for GRIN-related DEE (Amador et al., 2020; Bertocchi et al., 2021). Second, while a mutation affecting one allele in humans is sufficient to lead to DEEs, in animal models the phenotype is largely visible in homozygosity. Attempts have been made to generate preclinical models of mutations present in heterozygosity in humans, but mostly without success, such as for models carrying a loss of function mutations in K^+^ channel genes (Oyrer et al., 2018). Moreover, the presence, as well intensity, and frequency of seizures and other symptoms are highly influenced by the genetic background. Finally, even if less investigated, environmental conditions are known to influence disease expression, so that mutants often show traits inconsistent with the human phenotype (Singh et al., 2008).

Besides incomplete penetrance, genetically modified mouse or rats displaying spontaneous epilepsy are rare, considering that they are often either not viable or die in the first days of life. The few viable models that show spontaneous seizures are associated with a high mortality rate and, given that seizure onset and frequency is highly unpredictable, they are difficult to study. Despite this, rodents with spontaneous seizures can still prove to be excellent preclinical tools such as, for example, mutant mice in which the Tsc1 gene was deleted in Emx1-expressing embryonic telencephalic neural stem cells (NSCs), which recapitulate Tuberous Sclerosis Complex (TSC) neuropathological lesions and present spontaneous generalized cortical seizures in 100% of the mutants by postnatal day (PND) 13 (Magri et al., 2011). Notably, the delivery of chronic rapamycin treatment since PND 8 every other day resulted in increased body weight, no seizures and mortality, and reduced anxiety- and depression-like phenotype (Cambiaghi et al., 2013b).

Most preclinical rodent models exhibit reduced thresholds to electrically or pharmacologically induced seizures compared to wild-type littermates, while a minority show reflex epilepsy, meaning that the recurring crisis is provoked by clearly identifiable triggers or stimuli, mimicking the “two-hit” hypothesis (Love, 2005; Faingold, 2012; Spagnoli et al., 2015). A significant portion of this last group shows, in particular, generalized seizures when exposed to a specific acoustic stimulus, namely audiogenic seizures (AGS). AGS is generally induced by an aversive sound with a specific frequency and intensity, to which rodents manifest a stereotyped response that includes, in order: wild running, loss of stability with tonic–clonic crisis, and tonic seizure with limbs extension towards the tail, eventually followed by death from respiratory arrest (AGS-RA; Faingold, 2012). AGS susceptibility usually diminishes with age, as in DBA/1 and DBA/2 mice. These classic inbred strains are commonly used to study sudden unexpected death in epilepsy (SUDEP), given their susceptibility to AGS-RA (Pansani et al., 2016). However, this type of reflex epilepsy is also found in adult rodents, even in models of human syndromes with spontaneous seizures. In the synapsin triple-knockout (Syn-TKO) mice, for example, the seizure susceptibility follows a temporal evolution in which the seizure-provoking maneuver has a peak at about 2 months of age and then rapidly declines (Cambiaghi et al., 2013a). Despite the inconsistency with the human condition, these animals allow for rapid and reliable analysis of the anticonvulsant properties of new antiseizure medications (ASM) or therapeutics. A similar example is the GluN2A(N615S)-expressing mice, carrying a point mutation analogous to those found in children affected by DEE. GluN2A(N615S) mice show high penetrance to AGS-RA (Bertocchi et al., 2021) and no reduction in susceptibility in adult ages, thus allowing the preclinical assessment of specific antiepileptic treatments at different developmental stages and the possibility to study the mechanisms involved in SUDEP. This feature is difficult to observe in other animal models and opens experimental possibilities impossible to achieve with clinical studies.

In conclusion, preclinical rodent models may not faithfully reproduce the human symptom spectrum, even when carrying the same mutation found in patients. Importantly, however, they offer the unique possibility to study not only epileptic activity but also pathways and disease mechanisms associated with it and related neuropsychiatric impairments that cannot be reliably investigated in other models (Figure 1F).

### 2.2. E/I balance and activity-dependent plasticity: Two important therapeutic targets

Epilepsy is a network disease involving the development of complex hyperexcitable networks and brain-wide circuits to generate seizures (Englot et al., 2015; Rao and Lowenstein, 2015; Dong et al., 2016; Geier and Lehnertz, 2017; Pfisterer et al., 2020). Early deficits in neuronal activity and connectivity contribute to a developmental cascade affecting brain organization (Lewis et al., 2017) and different interacting brain regions are likely involved in generating E/I imbalance (Rao and Lowenstein, 2015; Shao et al., 2019) for the initiation and propagation of epilepsy (Figure 1). Converging evidence also demonstrates that an imbalance in the E/I ratio in various brain regions is crucially involved in functional deficits at the neural network level, leading to abnormal behavior in different neurodevelopmental disorders (NDDs), such as autism and attention deficit hyperactivity disorder (ADHD), and representing, therefore, a key therapeutic target (Tang et al., 2021). Accordingly, anomalies in E/I balance underlie cerebral response deficits, hyperresponsiveness, and amplified cortical responses to sensory stimuli (Sohal and Rubenstein, 2019). EEG abnormalities and defects in the synchronization and communication of cortical networks are frequently linked with decreased cognitive function and sociability, hyperactivity, impulsivity, and repetitive, stereotyped behaviors (Razak et al., 2021).

Synaptic and circuit E/I balances are established and fine-tuned during brain development (Lee et al., 2017). A substantial portion of the primary pathological changes in this equilibrium occurs during embryonic or early postnatal periods and exerts long-lasting effects. However, the cellular and network-specific changes that characterize the E/I imbalance are still poorly understood, highlighting the importance of pursuing detailed and integrative analyses of E/I imbalance in animal models, particularly during perinatal and postnatal critical periods.

Preclinical models are essential for studying E/I imbalance and age-related changes in brain circuits due to activity-dependent plasticity that likely develops over time. For example, during juvenile development, the experience-dependent formation of a specialized sheath made of extracellular matrix components around a portion of neurons, named “perineuronal net” (PNN), marks the closure of critical periods of neuronal plasticity (Carulli et al., 2010). Interestingly, the formation of PNNs happens in concomitance with the well-known developmental switch in the expression of NMDAR GluN2B/A subunits, increasing the system “maturity” particularly in higher brain structures (Acutain et al., 2021). Moreover, it has been suggested that extracellular sulfated proteoglycans, enriched in PNN, play an exquisite role in regulating intracellular chloride ion concentrations in neurons and, consequently, the direction of inhibitory/excitatory GABA responses (Glykys et al., 2014). Not surprisingly, therefore, with GABAergic signaling, PNNs are frequently altered in animal models for different NDDs, leading to E/I imbalance and hyperexcitability of local cortical circuits. For example, the hyperexcitability of auditory cortex pyramidal neurons in mouse models of fragile X syndrome (Fmr1KO mice) is well described to lead to hyper-responsivity to sound (Rotschafer and Razak, 2013). Here, pharmacologically or environmentally induced E/I balance and PNN readjustment have rescued hyperresponsivity and susceptibility to AGS (Lovelace et al., 2016, 2020; Pirbhoy et al., 2020).

Fragile X Mental Retardation Protein (FMRP1) loss, as the effects of other gene mutations, may alter neuronal excitability and plasticity in opposite directions in different brain areas, generating heterogeneous regional and temporal effects on synaptic E/I balance to modulate neuronal firing (Lee et al., 2017). The allostatic establishment of excessive activation of glutamatergic neurons may impair plasticity, brain functions, physiology, and behavior. It would be, therefore, crucial to selectively target and dampen the activity of hyper-excitable neurons involved in these abnormalities. Understanding the dynamics of E/I balance-related alterations in brain circuitry that subserves cognitive and behavioral functions and the developmental refinements of the altered circuitry during critical periods of plasticity could provide novel insights into the nature of different NDDs.

### 2.3. Importance of video-EEG recording in seizure monitoring

Preclinical models represent a necessary step in developing new treatments for epilepsy and comorbidities. In addition to tolerability, the identification of ASM effectiveness is mainly based on its effects on seizure threshold or their spreading and, more generally, on behavior (Swinyard and Kupferberg, 1985). When studying the (chronic) effects of possible ASM, the combination of time-locked video and EEG recording (usually called video-EEG monitoring) and different behavioral tests can be considered the best option. Video-EEG allows a precise correlation between a given event and brain activity (superficial or deep). Off-line analysis can be helpful to confirm the association between a specific behavior and an epileptic seizure or a different episode (such as scratching, movement disorder, artifacts, or other false positives, for example, grooming; Cambiaghi et al., 2013b). Moreover, video-EEG monitoring is essential to classify seizures and determine their temporal dynamics. Lastly, besides qualitative observation, quantitative EEG data analysis can be associated with a particular behavior or task, such as sleep or a cognitive test (for an exhaustive review, see Cambiaghi et al., 2015).

### 2.4. Circuit-based innovative rAAV tools for therapy

**Figure 2:**
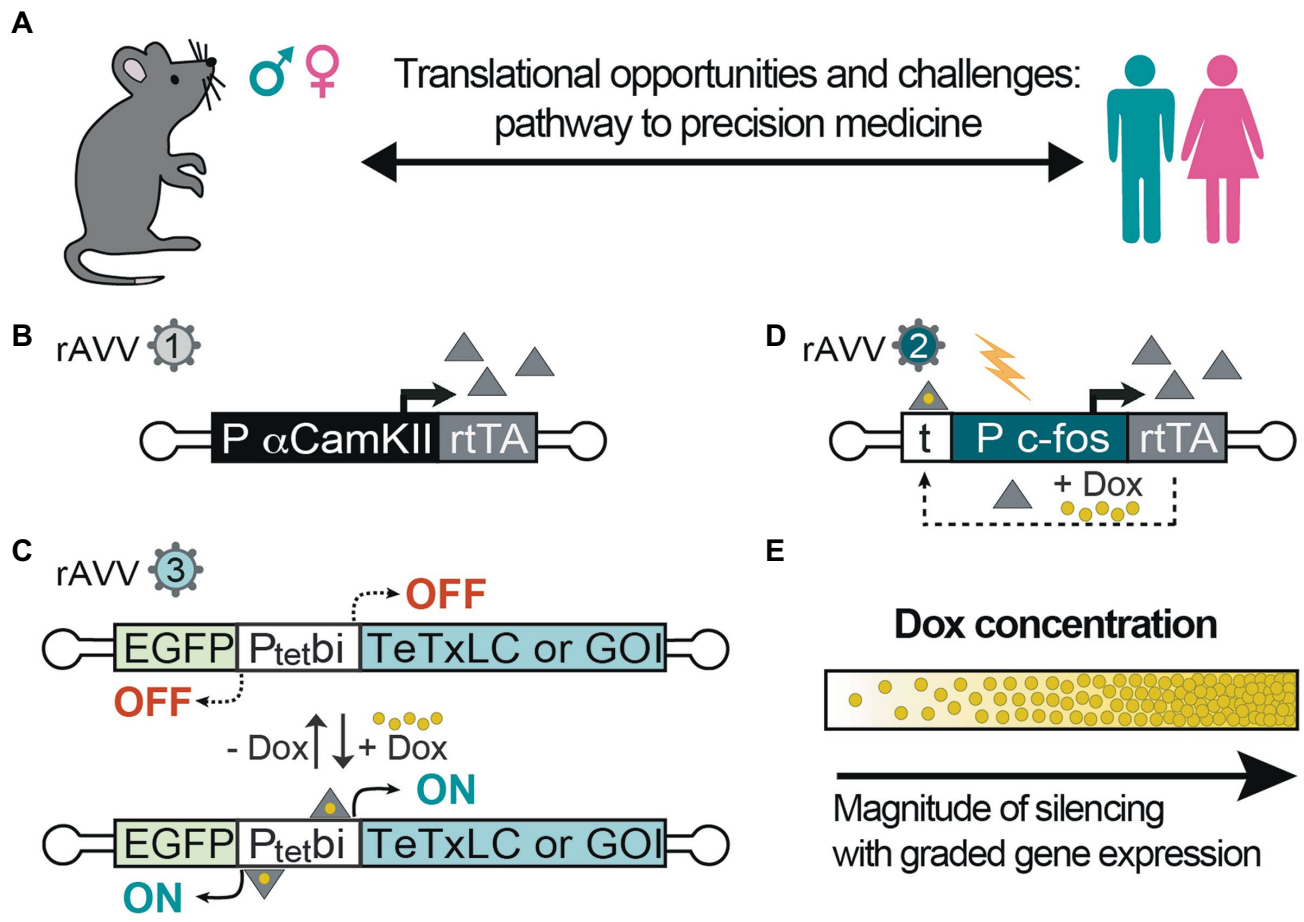
Inducible control of excitation-inhibition balance and gene activity. **(A)** Animal models are useful for testing specific hypotheses-driven experiments, also regarding gender differences in various pathologies and in response to therapy. Thus far, they represent a powerful approach to move preclinical studies into clinical trials; **(B)** Recombinant adeno-associated virus (rAAVs) can be equipped with α-calmodulin kinase-2 promoter (αCaMK2) to express reverse tetracycline transactivator (rtTA; rAAV1), which upon **(C)** binding to the Ptetbi promoter (rAAV3) in the presence of doxycycline (Dox) activate the expression of tetanus toxin light chain (TeTxLC) to block evoked synaptic transmission in excitatory neurons. In the absence of Dox, the expression is switched-off and synaptic transmission can resume; **(D)** With another system based on c-fos promoter with upstream tetracycline operators (t), hyperactive cells are specifically targeted in the presence of Dox to express rtTA, which establishes an autoregulated loop for rtTA-dependent rtTA expression, thereby E/I can be controlled in the tagged cells using TeTxLC or any gene of interest (GOI) as in **(C)**. Indeed, these technologies shown here **(B/C)** or **(D/C)** can also be used to express any gene of interest to override the mutations responsible for epilepsy. **(E)** Moreover, the level of expression and synaptic silencing can be controlled at different concentrations of Dox. Controlled levels of gene expression can allow for proper titration of E/I balance.

Animal models can be rapidly generated, and they are valuable tools to investigate neurobiological processes across brain regions *in vivo* and in acute brain slices and would help provide prospects for precision medicine in humans to treat epilepsies (Figure 2A). With the advent of recombinant adeno-associated viruses (rAAVs) delivery for local targeting of brain circuits (Dogbevia et al., 2016) and intravascular delivery for brain-wide-targeting (Goertsen et al., 2022), it is possible to treat or perhaps even cure these disorders. Specific brain circuits can be targeted and manipulated by inducible gene expression systems using rAAVs to control plasticity (Hasan et al., 2013) and E/I balance (Reus-García et al., 2021). Introducing normal gene(s) to brain cells to overcome the negative effects of the mutated gene(s) is becoming a feasible approach (Murphy et al., 2013; Dogbevia et al., 2016; Figures 2B,C). It is also possible to selectively target hyperactive neurons using the c-fos-equipped genetic switches (Hasan et al., 2019; Keller et al., 2022; Figures 2B,D). These inducible switches can be controlled by different levels of the chemical inducer provided to the animals (and potentially humans) to control graded levels of gene expression for proper titration of E/I balance (Figure 2E) and effective recovery of mutant gene dysfunctional activities.

## 3. Discussion

DEEs encompass a wide range of highly multifaceted syndromes, predominantly of genetic origin, with the genes involved mainly coding for ion channels or proteins controlling neuronal excitability. Patients with DEEs display severe symptoms and high heterogeneity, even if carrying the same mutation. Additionally, the genetic background, plasticity, and epigenetic mechanisms significantly impact mutation expressivity (Guerrini et al., 2023). This variability adds complexity to uncovering the correlation between genotype and phenotype and the development of effective therapies. Preclinical models constitute an invaluable tool for better understanding the multifaceted pathophysiology of various DEEs and for studying the effect of candidate drugs. Knock-in mice reproduce more faithfully the unique human disorders; however, many DEE syndromes still lack comprehensive genetic models.

A key aspect of studying candidate ASM effects in animal models is linked to the possibility of seizure characterization and the analysis of background EEG abnormalities and their correlation with behavior.

EEG abnormalities *per se* can have a detrimental effect on the developing brain, and the results that a candidate ASM may have on EEG in animal models can provide valuable insight into how much clinical improvement can be obtained by treatment-induced EEG modulation. We highlight the importance of adopting time-locked video-EEG recordings to detect the changes and synchronization of different brain areas’ activity and their correlation with specific/abnormal behaviors.

Preclinical and clinical studies suggest that the high-fat, low-carbohydrate ketogenic diet (KD) effectively controls drug-resistant seizures (Nei et al., 2014; Ruan et al., 2022). Evidence also demonstrated some efficacy in improving cognitive abilities (Rho and Boison, 2022; Zhu et al., 2022). However, such dietary therapy is difficult to adhere to, needs continuous monitoring, is unsuitable for all patients, and has several side effects (Rho and Boison, 2022; Zhu et al., 2022; Operto et al., 2023). A better understanding of the molecular mechanisms through which KD and novel ASM can alleviate seizures and cognitive deficits is fundamental for developing new drugs with an overall efficacy while avoiding side effects as much as possible. Animal models are well suited for this purpose; they offer the advantage of studying pathways relevant to the disease pathology, as often the principal target (i.e., a defective gene and encoded protein) is not druggable.

The search for effective treatment for various forms of DEE is a priority for science and society. In recent years, a drive for precision medicine using new candidate ASM and targeted gene therapy has emerged as a viable treatment strategy for different diseases and holds much promise for drug-refractory epilepsies. Further studies, both preclinical and in children suffering from resistant seizures, are needed. Considering the variability and phenotypic pleiotropy typical of DEEs, genetic therapeutic approaches able to precisely target common pathological mechanisms, such as the E/I imbalance, might open prospects for these devastating syndromes and more common types of epilepsy. With large-scale changes in brain networks during early disease onset, developing effective therapies at neonatal or even prenatal stages would be imperative to treat these debilitating disorders.

In conclusion, there is a great need for novel preclinical models for the different forms of DEEs, considering their potential to assess and profile the effectiveness of therapeutic strategies aimed at contrasting not only the epileptic phenotype, but also the wide range of comorbidities experienced by the patients, from behavioral to cognitive deficits, despite the difficulties in transferring the results from preclinical to clinical practice.

## Author contributions

IB conceived and wrote the first draft. All authors contributed to the article and approved the submitted version.

## Funding

This work was supported by Dipartimento di Neuroscienze Rita Levi Montalcini, Università di Torino, Fondi Ricerca Locale 2021 (BERI_RILO_21_01). We thank Fondazione CRT 2019 (RF=2019.2285) and Fondi Ricerca Locale, Dipartimento di Neuroscienze Rita Levi Montalcini, Università di Torino for IB and the ERA-NET NEURON (TopDown PTSD; MH), Grant PCIN-2017-120 MCIN/AEI/10.13039/501100011033/ERDF/NextGenerationEU/PRTR (MH), Grant PID2021-124013OB-I00 MCIN/AEI/10.13039/501100011033 (MH), RTI2018-101624-B-I00 (MH), National Institute of Health (MH).

## Conflict of interest

The authors declare that the research was conducted in the absence of any commercial or financial relationships that could be construed as a potential conflict of interest.

## Publisher’s note

All claims expressed in this article are solely those of the authors and do not necessarily represent those of their affiliated organizations, or those of the publisher, the editors and the reviewers. Any product that may be evaluated in this article, or claim that may be made by its manufacturer, is not guaranteed or endorsed by the publisher.
